# Plastome structure, phylogenomics and evolution of plastid genes in *Swertia* (Gentianaceae) in the Qing-Tibetan Plateau

**DOI:** 10.1186/s12870-022-03577-x

**Published:** 2022-04-12

**Authors:** Qian Cao, Qingbo Gao, Xiaolei Ma, Faqi Zhang, Rui Xing, Xiaofeng Chi, Shilong Chen

**Affiliations:** 1 Key Laboratory of Crop Molecular Breeding of Qinghai Province, Northwest Institute of Plateau Biology, Chinese Academy of Sciences, Xining, 810008 China; 2 University of Chinese Academy of Sciences, Beijing, 100049 China

**Keywords:** *Swertia*, Plastome, Pseudogene, Positive selection, Polyphyly, Phylogenetic relationships, Rapid radiation

## Abstract

**Background:**

The genus *Swertia* is of great medicinal importance and one of the most taxonomically challenging taxa within Gentianaceae, largely due to the morphological similarities of species within this genus and with its closely related genera. Previous molecular studies confirmed its polyphyly but suffered from low phylogenetic resolutions because only limited sequence loci were used. Thus, we conducted the structural, gene evolutionary, and phylogenetic analyses of 11 newly obtained plastomes of *Swertia*. Our result greatly improved the phylogenetic resolutions in *Swertia*, shed new light on the plastome evolution and phylogenetic relationships of this genus.

**Results:**

The 11 *Swertia* plastomes together with the published seven species proved highly similar in overall size, structure, gene order, and content, but revealed some structural variations caused by the expansion and contraction of the IRb region into the LSC region, due to the heterogeneous length of the ψ*ycf1*. The gene *rps16* was found to be in a state flux with pseudogenes or completely lost. Similar situation was also documented in other genera of Gentianaceae. This might imply loss of the gene in the common ancestor of Gentianaceae. The distribution plot of ENC vs. GC3 showed all these plastomes arranging very close in the Wright line with an expected ENC value (49–52%), suggesting the codon usage of *Swertia* was mainly constrained by a GC mutation bias. Most of the genes remained under the purifying selection, however, the *cemA* was identified under positive selection, possibly reflecting an adaptive response to low CO_2_ atmospheric conditions during the Late Miocene. Our phylogenomic analyses, based on 74 protein-coding genes (CDS), supported the polyphyly of *Swertia* with its close allies in the subtribe Swertiinae, presumably due to recent rapid radiation. The topology inferred from our phylogenetic analyses partly supported the current taxonomic treatment. Finally, several highly variable loci were identified, which can be used in future phylogenetic studies and accurate identification of medicinal genuineness of *Swertia*.

**Conclusions:**

Our study confirmed the polyphyly of *Swertia* and demonstrated the power of plastome phylogenomics in improvement of phylogenetic resolution, thus contributing to a better understanding of the evolutionary history of *Swertia*.

**Supplementary Information:**

The online version contains supplementary material available at 10.1186/s12870-022-03577-x.

## Background

The genus *Swertia* Linnaeus, belonging to subtribe Swertiinae, family Gentianaceae with reported 168 species, is widely distributed among Asia, Africa, North America, and Europe [1]. Seventy-nine species of *Swertia* are distributed over China [2], with a diversification center in the Qing-Tibetan Plateau region, especially in south-western China and the Himalaya region [1–3]. Ho described *Swertia* in China and classified them under seven sections [2]. Later, based on their previous studies, Ho and Liu published a worldwide classification of *Swertia*, comprising 168 species, under three subgenus, 11 sections [1].

*Swertia* can be recognized with rotated flowers, one or two nectaries per petal lobe, and a variety of different appendages around the nectaries. However, this genus is one of the most taxonomically difficult genera within Gentianaceae, largely due to the morphological similarities (i.e., nectariferous and rotate corolla lobes) of the species in *Swertia* and with its allies, namely, *Halenia*, *Lomatogonium*, *Lomatogoniopsis* and *veratrilla* [1, 4–7]. Over the past decades, continues efforts have been devoted to molecular phylogeny studies of *Swertia* based on ITS and plastid sequence loci. It has become clear that *Swertia* was highly polyphyletic, intertwining with other genera in the subtribe Swertiinae on the inferred trees. Some clades were left unresolved and usually with weak node support, e.g., the *Comastoma*-*Lomatogonium*-sect. *Platynema*, *Halenia*-sect. *Swertopsis*, most likely due to only a few molecular markers were employed [4–6, 8]. More recently, a study based on 76 protein-coding genes generated from plastomes, supported *Swertia’s* polyphyly with greatly improved resolution when compared with those earlier molecular studies, however, only a few *Swertia* species were included as the representative of this genus [9].

Species of *Swertia* have long been used in traditional herbal medicine in Asian countries, including China, India, Korea, and Japan. The crude drug of *Swertia* is applied to treat a variety of diseases, including hepatic, choleric, inflammatory diseases, and bitter stomachic [10–12]. These herbaceous plants are rich in xanthones, flavonoids, iridoid and seco-iridoid glycosides, terpenoids, and alkaloids [13]. Despite the excellent medicinal value and abundant medicinal studies, the accurate identification of genuineness of *Swertia* species has been proved difficult and still lacks of DNA barcoding method [14]. To this aim, barcoding by a comparative plastome method of *Swertia* authentication is needed to assure good medicinal quality.

In almost all land plants, plastomes are highly conserved in structural organization, gene arrangement, and gene content [15–17]. A typical plastome usually has a quadripartite structure of a large single copy region (LSC) of 82–90 kb, a small single copy (SSC) of 15–20 kb, and two identical copies of inverted regions (IRa & IRb) [17]. In photosynthetic angiosperms, most plastomes contain 110–133 distinct genes, including approximately 80 protein-coding genes (CDS), 30 transfer RNA (tRNA) genes, and four ribosomal RNA (rRNA) genes [17]. Due to their conserved structure, lack of recombination, and uniparental inheritance [15, 18], plastomes data have recently been employed to generate highly resolved phylogenies or to efficiently barcode and identify plant species, especially in taxonomically complex groups [19–23].

Here, we report newly sequenced 11 plastomes of *Swertia*. Our aims were to: (a) infer the plastome structural evolution and adaptation of *Swertia*; (b) identify the most variable regions as candidate DNA barcodes for future *Swertia* species identification; (c) test the phylogeny of Swertia in regard to its current taxonomic treatment.

## Results

### Plastome features of *Swertia*

For the 11 newly sequenced *Swertia* species, Illumina pair end sequencing generated 13,182,812 (*S. dichotoma*) to 60,069,018 (*S. przewalskii*) raw reads. After quality filtering, 13,167,950 (*S. dichotoma*) to 59,482,032 (*S. przewalskii*) clean reads were obtained. The assembled plastomes of the 11 *Swertia* species had coverage depth from 760 X (*S. dichotoma*) to 7916 X (*S. cincta*) (Additional file 9, Fig. S1). The raw sequencing data were deposited in the NCBI GenBank (Additional file 1, Table S1).

**Fig. 1:**
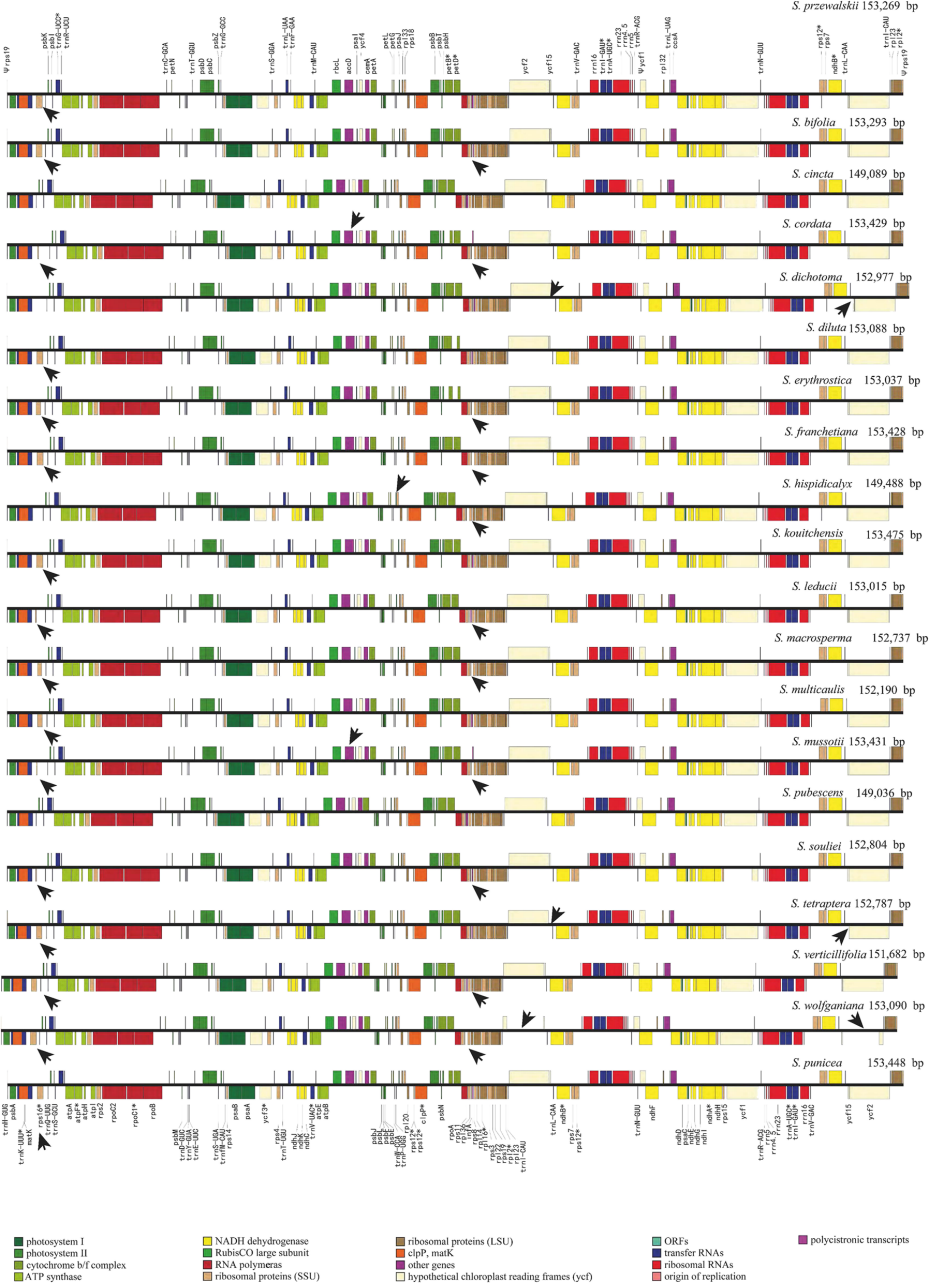
Plastomes maps of *Swertia.* Black arrows indicated pseudogenes other than ψ*ycf1* and ψ*rps19*

The lengths of the 20 plastomes varied from 149,036 (*S. pubescens*) to 154,073 bp (*S. kouitchensis*) (Fig. 1, Table 1). All the 20 *Swertia* plastomes contained two copies of IR regions, each ranging from 25,331 (*S. cincta*) to 25,889 bp (*S. cordata*). The IRs were separated by a LSC region ranging from 83,610 (*S. cordata*) to 80,432 bp (*S. pubescens*) and a SSC region ranging from 17,903 (*S. hispidicalyx*) to 18,547 bp (*S. tetraptera*). The overall GC content of the 20 *Swertia* plastomes were similar and ranged from 38% in *S. tetraptera* to 38.2% in *S. cincta*, *S. macrosperma*, and *S. pubescens* (Table 1).

**Table 1:** Summary of 20 *Swertia* plastomes

Species	Plastome size (bp)	LSC	IRs	SSC	GC (%)	CDS	tRNA	rRNA
*S. bifolia*^a^	153,293	83,574	25,759	18,201	38.1	85 (7)	37 (7)	8 (4)
*S. cincta*^a^	149,089	80,481	25,331	17,946	38.2	86 (7)	37 (7)	8 (4)
*S. cordata*	153,429	83,610	25,889	18,039	38.1	84 (7)	37 (7)	8 (4)
*S. dichotoma*^a^	152,977	83,044	25,815	18,303	38.1	85 (7)	37 (7)	8 (4)
*S. diluta*^a^	153,088	83,310	25,792	18,362	38.1	86 (7)	37 (7)	8 (4)
*S. erythrosticta*	153,037	83,370	25,709	18,249	38.1	85 (7)	37 (7)	8 (4)
*S. franchetiana*^a^	153,428	83,564	25,761	18,342	38.1	84 (7)	37 (7)	8 (4)
*S. hispidicalyx*	149,488	80,727	25,429	17,903	38.1	84 (7)	37 (7)	8 (4)
*S. kouitchensis*^a^	153,475	83,595	25,766	18,348	38.1	86 (7)	37 (7)	8 (4)
*S. leducii*	153,015	83,048	25,786	18,395	38.1	85 (7)	37 (7)	8 (4)
*S. macrosperma*^a^	152,737	83,046	25,730	18,231	38.2	85 (7)	37 (7)	8 (4)
*S. multicaulis*	152,190	82,893	25,477	18,343	38.1	86 (7)	37 (7)	8 (4)
*S. mussotii*	153,431	83,567	25,761	18,342	38.1	84 (7)	37 (7)	8 (4)
*S. przewalskii*^a^	153,269	83,507	25,781	18,200	38.1	86 (7)	37 (7)	8 (4)
*S. punicea*^a^	153,448	83,535	25,784	18,345	38.1	86 (7)	37 (7)	8 (4)
*S. pubescens*^a^	149,036	80,432	25,334	17,936	38.2	86 (7)	37 (7)	8 (4)
*S. souliei*	152,804	83,195	25,756	18,105	38.1	85 (7)	37 (7)	8 (4)
*S. tetraptera*	152,787	83,093	25,759	18,236	38.0	85 (7)	37 (7)	8 (4)
*S. verticillifolia*	151,682	82,623	25,362	18,335	38.1	85 (7)	37 (7)	8 (4)
*S. wolfangiana*^a^	153,090	83,374	25,735	18,246	38.1	85 (7)	37 (7)	8 (4)

^a^The newly generated plastomes

The 20 *Swertia* plastomes contained 129–131 genes, composing of 84–86 protein-coding genes (CDS), 8 rRNAs and 37 tRNAs (Fig. 1, Table 1). Twelve CDSs and six tRNAs contained one intron. The *clpP* and *ycf3* contained two introns (Table 2). The intron of *trnK*-*UUU* included the coding sequence for the *matK*. *Rps12* was identified as a trans-spliced gene, with the first exon (5′ end of the sequence) in the LSC region (*rpl20_clpP*), and the remaining two exons (3′ end of the sequence) in the IR region (*rps7_trnV*-*GAC*). *Ycf1* sequence was duplicated in all the 20 *Swertia*, with one complete copy (1,014–5,517 bp) located on the SSC-IRa border served as a functional gene, and one incomplete copy (920–1,008 bp) on the IRb-SSC border identified as a pseudogene. The same situation was also found in *rps19*, one complete copy (279 bp) located on the LSC-IRb border acted as a functional gene, while the incomplete copy (161–303 bp) on the Ira-LSC border identified as a pseudogene. ψ*ycf1* and ψ*rps19* were resulted from incomplete duplications of their functional copes. Furthermore, *rps16* was either identified as a pseudogene (in 16 species) or completely lost (in four species). Also, ψ*infA* were observed in 11 *Swertia* plastomes. In addition, ψ*accD* were found in *S. cordata* and *S. mussotii*, ψ*rpl33* in *S. hispidicalyx*, and Ψ*ycf2* in *S. wolfganiana* (Fig. 1, Table 3).

**Table 2:** Genes annotated in 20 *Swertia* plastomes

Gene products of the 20 *Swertia* plastomes	
Photosystem I	*psaA, B, C, I, J*
Photosystem II	*psbA, B, C, D, E, F, H, I, J, K, L, M, N, T, Z*
Cytochrome b6_f	*petA, B(1), D(1), G, L, N*
ATP synthase	*atpA, B, E, F(1), H, I*
Rubisco	*rbcL*
NADH oxidoreductase	*ndhA(1), B(1) (3), C, D, E, F, G, H, I, J, K*
Large subunit ribosomal proteins	*rpl2(1)(3), 14, 16(1), 20, 22, 23(3), 32, 33, 36*
Small subunit ribosomal proteins	*rps2, 3, 4, 7(3), 8, 11, 12 (1) (3), 14, 15, 16(1),18, 19(1) (3)*
RNA polymerase	*rpoA, B, C1(1), C2*
Unknown function protein-coding gene	*ycf1 (3), 2(3), 3(2), 4, 15 (3)*
Other genes	*accD, ccsA, cemA, clpP(2), infA, matK*
rRNAs	*rrn16(3), rrn23(3), rrn4.5(3), rrn5(3)*
tRNAs	*trnA-UGC(1)(3), trnC-GCA, trnD-GUC, trnE-UUC, trnF-GAA, trnG-GCC, trnG-UCC(1), trnH-GUG, trnI-CAU(3), trnI-GAU(1) (3), trnK-UUU(1), trnL-CAA(3), trnL-UAG, trnL-UAA(1), trnM-CAU, trnN-GUU(3), trnP-UGG, trnQ-UUG, trnR-ACG(3), trnR-UCU, trnS-GCU, trnS-UGA, trnS-GGA, trnS-UGA, trnT-GGU, trnT-UGU, trnV-GAC(3), trnV-UAC(1), trnW-CCA, trnY-GUA, trnfM-CAU*

(1) Gene containing a single intron

(2) gene containing two introns

(3) two gene copies in IRs

**Table 3:** Statistics of pseudogenes

Pseudogene	Species
*accD*	*S. cordata, S. mussotii*
*infA*	*S. bifolia, S. cordata, S. erythrosticta, S. franchetiana, S. hispidicalyx, S. leducii, S. macrosperma, S. mussotii, S. souliei, S. verticillifolia, S. wolfganiana*
*rpl33*	*S. hispidicalyx*
*rps16*	*S. bifolia, S. cordata, S. diluta, S. erythrosticta, S. franchetiana, S. kouitchensis, S. leducii, S. macrosperma, S. multicaulis, S. mussotii, S. souliei, S. przewalskii, S. punicea, S. tetraptera, S. verticillifolia, S. wolfganiana*
*rps19*	all the 20 *Swertia*
*ycf1*	all the 20 *Swertia*
*ycf15*	*S. dichotoma, S. tetraptera*
*ycf2*	*S. wolfganiana*

**Fig. 2:**
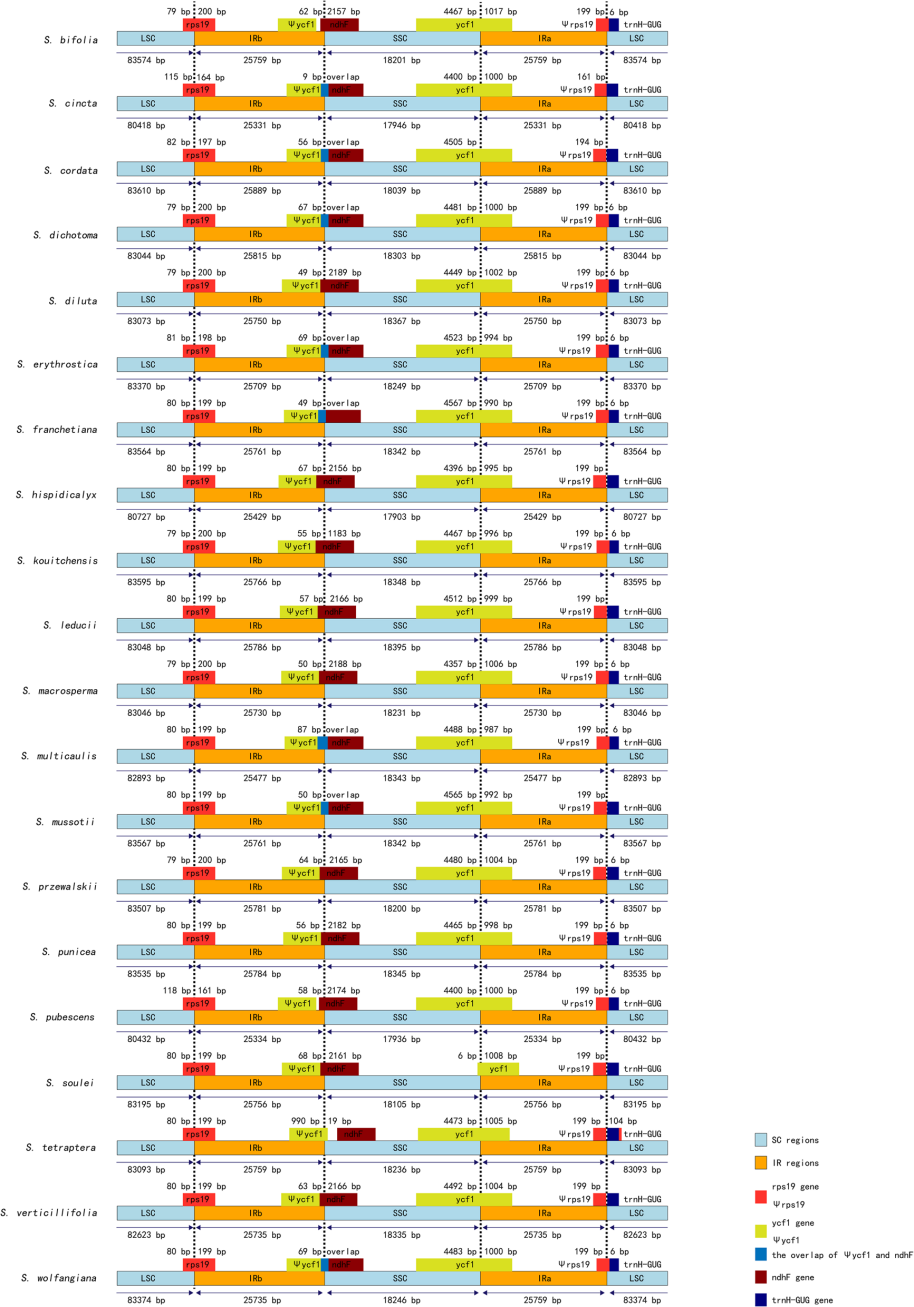
Comparison of LSC, IRs and SSC borders among 20 *Swertia* plastomes

The boundaries between IR and SSC/LSC regions in the 20 plastomes were compared (Fig. 2). The LSC/IRb junction was found only within the *rps19*. The Ira/SSC junction was found within the *ycf1*. The boundary between IRb and LSC slightly varied: 12 species were within the *ndhF* gene, and seven species within the overlapping region of the ψ*ycf1* and *ndhF*, only *S. tetraptera* was identified within the ψ*ycf1*. The Ira/LSC border was either within ψ*rps19* or adjacent to the *trnH*-*GUG*.

### Codon preference analysis

**Fig. 3:**
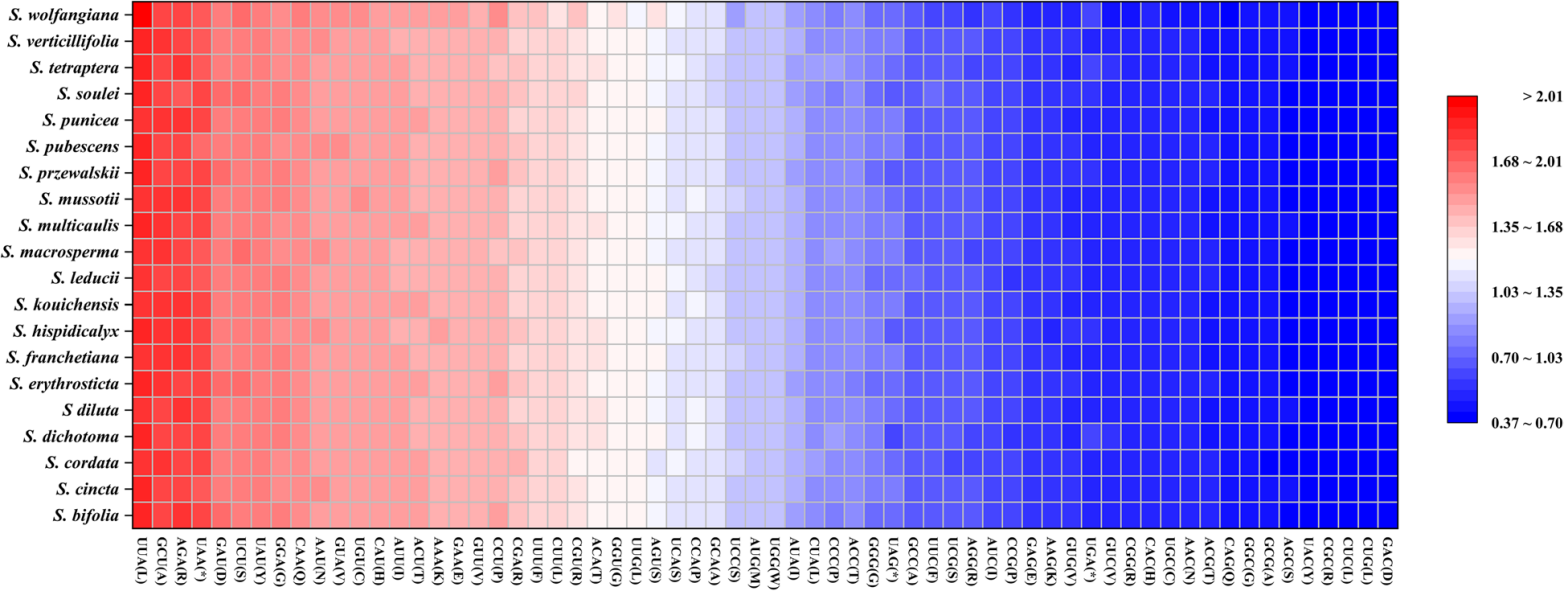
The RSCU values of all merged CDSs among 20 *Swertia* plastomes. Color key: the red values indicated higher RSCU values while the blue values indicated lower RSCU values

**Fig. 4:**
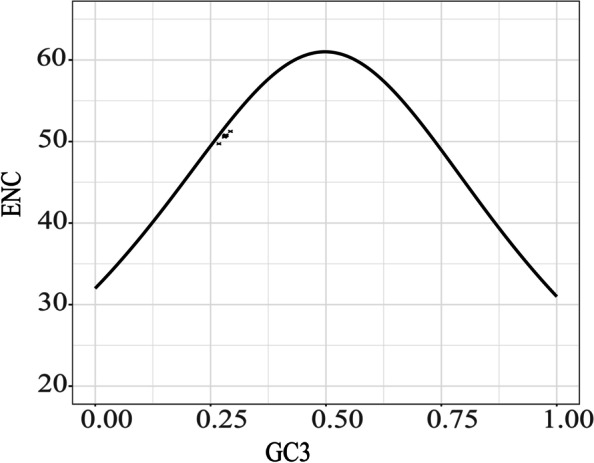
ENC-plot analysis

The total sequence sizes of the CDSs for codon analysis were 66,522–79,371 bp in the 20 *Swertia* plastomes, and they encoded 22,174–26,457 codons (Additional file 3, Table S3). Isoleucine (Ile) was encoded by the highest number of codons (1,901–2,223), whereas cysteine (Cys) was the least (261–308). The relative synonymous codon usage (RSCU) values of all codons in the form of a heatmap were shown in Fig. 3. The red indicated higher RSCU values and the blue indicated lower RSCU values. Thirty codons were used frequently with RSCU > 1. UUA showed a preference in all the 20 plastomes. All the biased 30 codons ended with a purine (A/U). The total GC content (GC, 0.383–0.398), GC content at first codon position (GC1, 0.456–0.463), GC content at second codon position (GC2, 0.384–0.391), and GC content at third codon position (GC3, 0.298–0.313) were less than 0.5, suggesting that the 20 plastomes tended to use A/T bases and A/T–ending codons. The codon adaptation index (CAI) values between 0.162 and 0.167 indicated a slight bias in codon usage in the 20 *Swertia* species. The values of the effective number of codons (ENC) ranged from 49.71 (*S. wolfangiana*) to 51.24 (*S. cordata*) (Table 4, Additional file 4, Table S4). Further, the ENC vs. GC3 plot (Fig. 4) showed the 20 *Swertia* plastomes clustered just below the Wright Curve[24, 25], and ranging around the expected ENC of 50% GC3.

**Table 4:** Codon feature of the 20 *Swertia* plastomes

Species	Codon No	GC1%	GC2%	GC3%	GCall%	ENC	CAI
*S. bifolia*	25,697	45.59	38.58	30.95	38.37	50.55	0.163
*S. cincta*	26,270	45.67	38.62	30.97	38.42	50.55	0.163
*S. cordata*	26,132	45.65	38.54	31.05	38.41	51.24	0.167
*S. dichotoma*	26,283	45.69	38.52	30.97	38.39	50.73	0.162
*S diluta*	26,266	45.65	38.64	31.20	38.49	50.74	0.162
*S. erythrosticta*	26,049	45.61	38.57	30.93	38.37	50.56	0.163
*S. franchetiana*	26,299	45.66	38.66	31.17	38.50	50.68	0.162
*S. hispidicalyx*	26,249	45.65	38.62	31.01	38.43	50.7	0.162
*S. kouichensis*	26,165	45.70	38.65	31.12	38.49	50.69	0.162
*S. leducii*	26,297	45.64	38.58	31.16	38.46	50.78	0.162
*S. macrosperma*	25,731	45.77	38.70	31.10	38.52	50.73	0.162
*S. multicaulis*	26,361	45.66	38.62	31.07	38.45	50.76	0.162
*S. mussotii*	25,635	45.75	38.70	31.15	38.53	50.68	0.162
*S. przewalskii*	26,340	45.61	38.58	30.94	38.38	50.58	0.163
*S. pubescens*	24,571	45.56	38.63	30.99	38.39	50.65	0.162
*S. punicea*	26,457	45.67	38.65	31.16	38.49	50.71	0.162
*S. souliei*	26,223	46.25	39.06	31.29	38.87	50.76	0.163
*S. tetraptera*	22,174	45.67	38.42	31.05	38.38	50.76	0.162
*S. verticillifolia*	25,697	45.67	38.58	31.09	38.45	50.77	0.162
*S. wolfangiana*	26,270	46.13	39.05	29.75	38.31	49.72	0.165

*GCall* the total GC content, *GC1* the GC content at first codon position, *GC2* the GC content at second codon position, *GC3* the GC content at third codon position, *CAI* the codon adaptation index, *ENC* the effective number of codons

### Characterization of repeat sequences and simple sequence repeats (SSR)

**Fig. 5:**
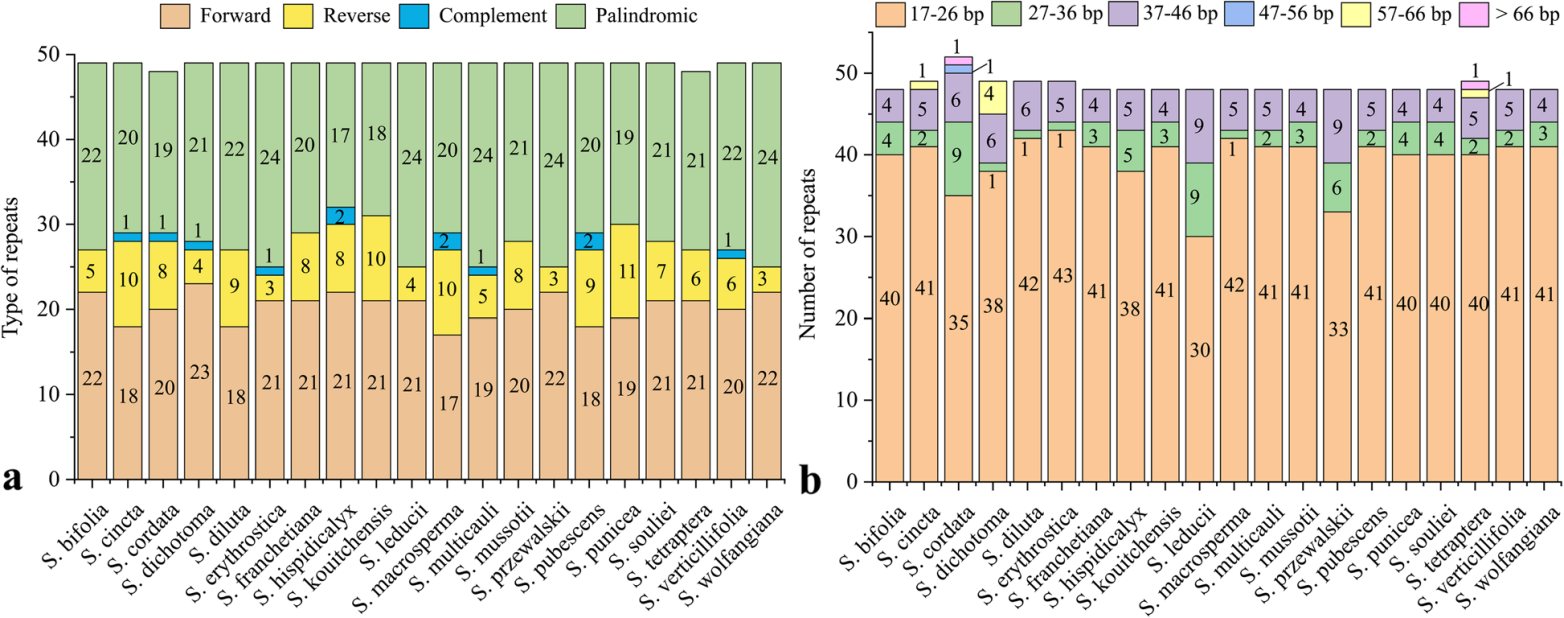
Analysis of repeat sequencies in the 20 *Swertia* plastomes. **a** Total number of four repeat types. **b** Number of repeats divided by length

In our study, a total of 980 repeat sequences were detected in the 20 *Swertia* plastomes, including four categories: forward, reverse, complement, and palindromic. The numbers of the four repeated types were as follows: 18–23 in forward repeats, 3–11 in reverse, 0–2 in complement, and 17–24 in palindromic (Fig. 5 a, Additional file 5, Table S5). The sizes of repeats ranged from 17 to 509 bp (Fig. 5 b, Table 5, Additional file 5, Table S5). According to the length of repeat sequences, we artificially divided them into six categories: 17–26 bp, 27–36 bp, 37–46 bp, 47–56 bp, 57–66 bp, and > 66 bp. Among them, the majority of repeats (81%) were 17–26 bp long, while only one repeat was found > 66 bp (509 bp forward repeat) in *S. cordata*. Slightly more than half (53.4%) of the repeat sequences were located in intergenic spacer (IGS).

**Table 5:** Repeat sequencies in the 20 *Swertia* plastomes

species	Forward	Reverse	Complement	Palindromic	length (bp)
*S. bifolia*	22	5		22	18–39
*S. cincta*	18	10	1	20	18–48
*S. cordata*	20	8	1	19	18–509
*S. dichotoma*	23	4	1	21	18–49
*S. diluta*	18	9		22	18–41
*S. erythrostica*	21	3	1	24	17–39
*S. franchetiana*	21	8		20	18–39
*S. hispidicalyx*	22	8	2	17	18–48
*S. kouitchensis*	21	10		18	18–39
*S. leducii*	21	4		24	18–48
*S. macrosperma*	17	10	2	20	18–48
*S. multicaulis*	19	5	1	24	18–48
*S. mussotii*	20	8		21	18–39
*S. przewalskii*	22	3		24	18–44
*S. pubescens*	18	9	2	20	18–48
*S. punicea*	19	11		19	19–39
*S. souliei*	21	7		21	18–39
*S. tetraptera*	21	6		21	18–48
*S. verticillifolia*	20	6	1	22	18–48
*S. wolfangiana*	22	3		24	18–39

A total of 588 simple sequence repeats (SSRs) were identified across the 20 *Swertia* plastomes (Table 6). The majority (94.4%) of these SSRs were monomers, particularly A/T, and their lengths were mostly in the 12–19 bp range. There were only 2.7% of dimers (AT and TA) and 2.9% of trimers (GAA, TTA, and ATT), no tetramers, hexamers, and pentamers were found.

**Table 6:** SSRs types in 20 *Swertia* plastomes

Species	A	C	G	T	AT	TA	GAA	TTA	ATT
*s. bifolia*	5	-	1	15	1	-	1	1	-
*S cincta*	13	-	-	10	-	-	-	-	-
*S. cordata*	18	1	-	22	1	1	-	-	-
*S. dichotoma*	16	2	-	15	-	3	-	-	-
*S. diluta*	9	2		14	-	-	-	-	1
*s. erythrostica*	5	2	1	16	1	-	-	1	-
*S. franchetiana*	11	1	-	18	-	-	-	-	1
*S. hisdicalyx*	15	1	-	14	1	-	-	-	-
*S. kouitchensis*	11	2	-	16	-	-	-	-	1
*S. leducii*	12	1	-	19	-	-	-	-	1
*S. macrosperma*	14	-	-	9	-	-	-	-	-
*S. multicaulis*	14	-	-	14	1	-	-	-	-
*S. mussotii*	12	1	-	17	-	-	-	-	1
*S. przewalskii*	6	1	-	13	1	-	1	1	-
*S. pubescens*	18	-	-	13	-	1	-	1	-
*S. punicea*	12	-	-	15	-	-	-	-	1
*S. souliei*	5	1	-	15	1	-	1	1	-
*S. tetraptera*	18	3	-	19	-	1	-	-	-
*S. verticillifolia*	15	1	-	13	2	1	-	-	1
*S. wolfangiana*	4	1	-	14	-	-	-	1	-

### Hypervariable regions

**Fig. 6:**
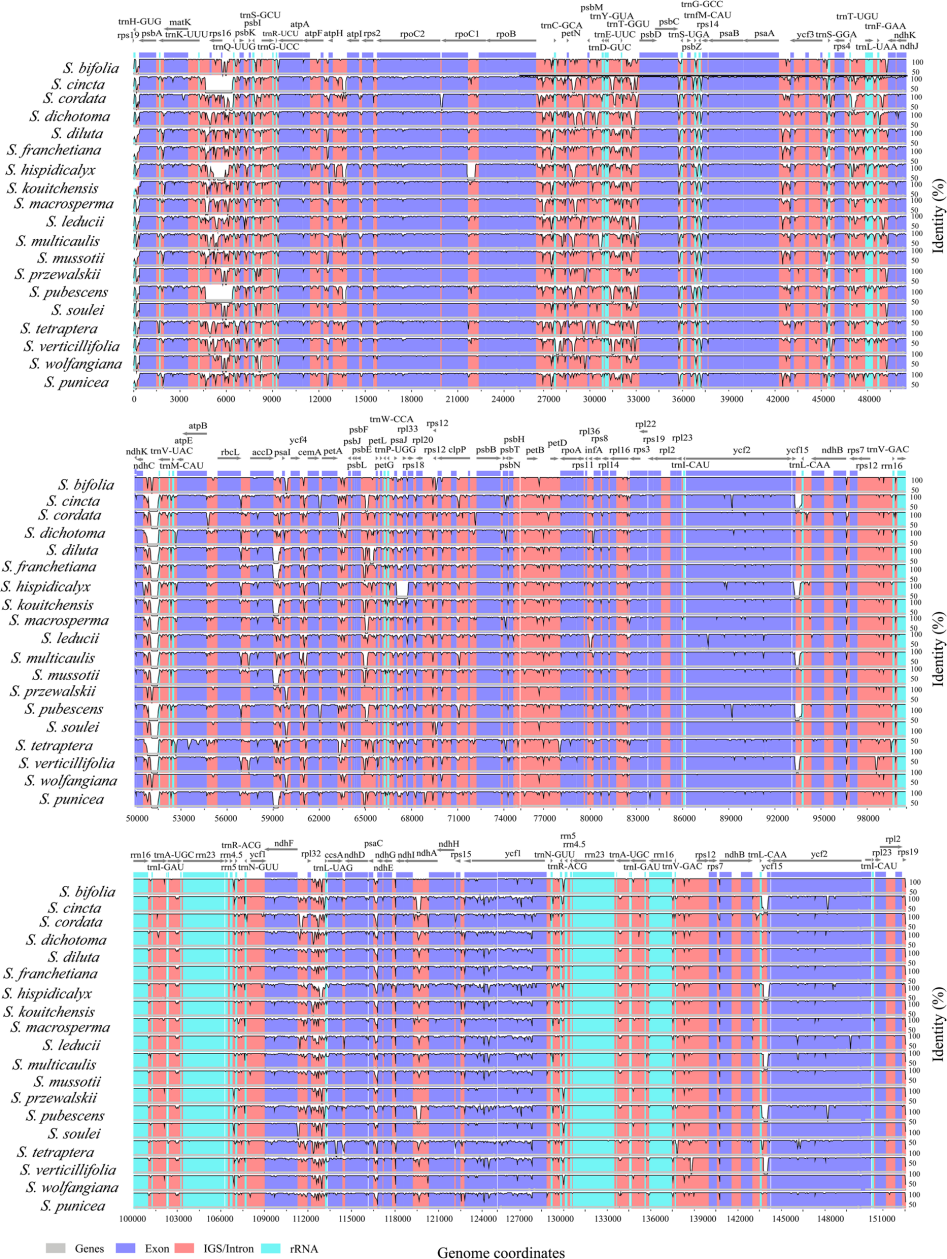
mVISTA-based sequence identity plot of 20 *Swertia* plastomes compared using *S. erythrosticta* as a reference. The top grey arrow shows genes in order (transcription direction) and the position of each gene. A 70% cut-off was used for the plots. The vertical scale represents the percentage of identity between 50 and 100%. Coding and non-coding regions (IGS/intron) are marked in purple and red, respectively, while conserved genes are in blue

**Fig. 7:**
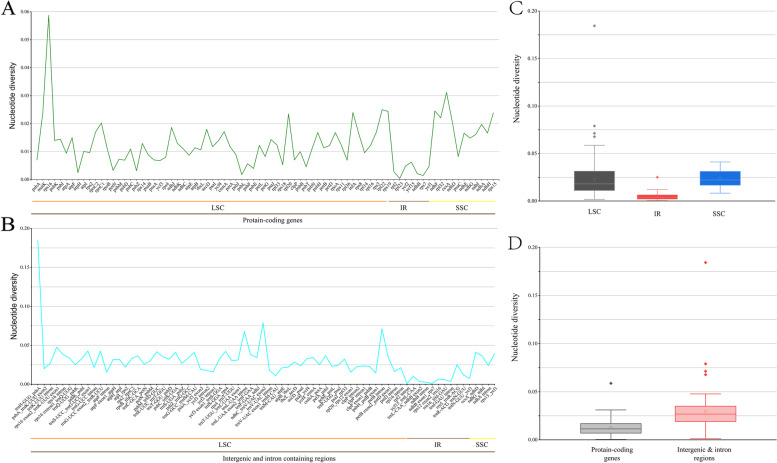
The genetic diversity based on Kimura’s two-parameter model. **a** The Pi values of protein-coding genes. **b** The Pi values of introns and intergenic regions. **c** Boxplots of Pi values difference among LSC, IR and SSC regions. **d** Boxplots of Pi values differences between protein-coding genes and intron & intergenic regions

Hypervariable regions were identified by genome-wide comparative analyses with *S. erythrostica* as the reference (Fig. 6). Our nucleotide diversity analyses included 79 CDSs (protein-coding regions) and 74 IGS and intron regions (non-coding regions) (Additional file 6, Table S6). The pairwise sequence divergence (Pi) values for each CDS ranged from 0.00035 (*rpl23*) to 0.05872 (*rps16*) with an average of 0.01271, in which 10 genes (*rps16*, *ccsA*, *rpl22*, *ndhF*, *rps19*, *matK*, *infA*, *rps15*, *rpl20*, *rpl32*) had remarkably high values (Pi > 0.02207). For the 74 non-coding regions, Pi values ranged from 0.00107 (*rpl2* intron) to 0.07895 (*ndhC_trnV*-*UAC*) with an average of 0.029679. Again, 10 of the non-coding regions (*ndhC_trnV*-*UAC*, *petB* intron, *trnL*-*UAA* intron, *rps16_trnK*-*UUU*, *trnL*-*UCC_trnG*-*UCC*, *trnG*-*UCC_trnR*-*UCU*, *trnS*-*GGA_rps4*, *psbM_trnD*-*GUC*, *trnG*-*GCC_trnfM*-*CAU*) had higher Pi values than the other regions (Fig. 7a, b). The non-coding regions exhibited more variation than the coding regions (Fig. 7c). The SSC region had the highest divergence and the IR regions had the lowest (Fig. 7d).

### Phylogenetic analysis

**Fig. 8:**
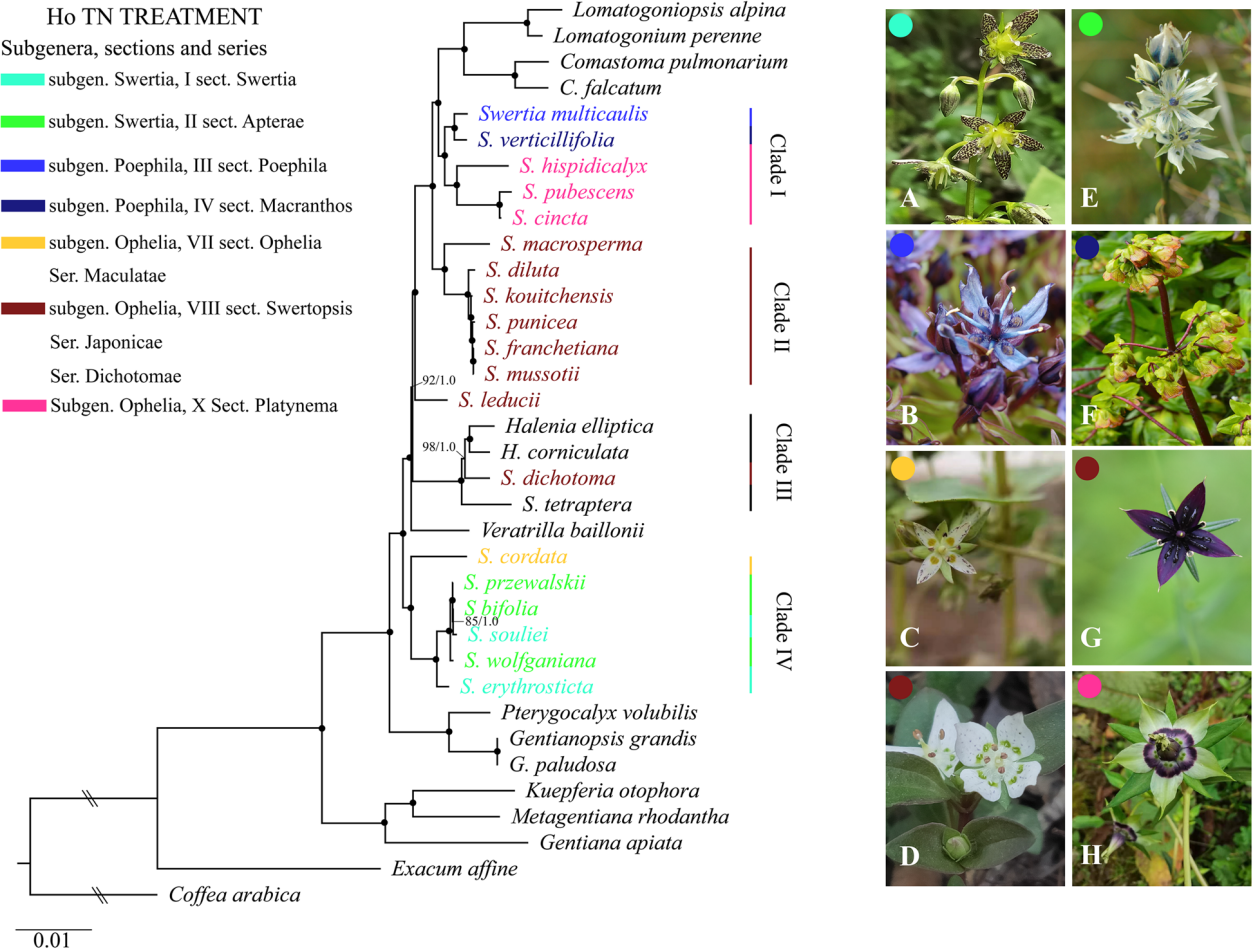
Phylogenic tree derived from 74 CDSs. The black spots indicate phylogenetic support values both from maximum likelihood and Bayesian with 100% bootstrap support (BS) and 1.00 posterior probability (PP) respectively. The double slashes indicate an artificial shortening of this branch, as the outgroup, *Coffer arabica*, produces a very long branch. The terminals are color-coded, with each color a clade in the phylogenic tree corresponding to a section in Ho TN taxonomic treatment, and a picture of species representing its section and/or series. The species displayed are A, *S. erythrosticta*; B, *S. multicaulis*; C, *S. cordata*; D, *S. dichotoma*; E, *S. przewalskii*; F. *S. verticillifolia*; G, *S. mussotii*; H, *S. cincta*. Photographs: Gao Qingbo, Ma Xiaolei, Cao Qian, Zhou Yubi, Xu Bo, Yang Fengmao

Both ML and BI analyses based on the 74 common CDSs shared between the 35 plastomes (20 *Swertia*, 10 Swertiinae, four Gentianinae, and one outgroup species) (Additional file 2, Table S2) inferred completely identical topologies with high support values (Fig. 8). The monophyly of both subtribe Swertiinae and subtribe Gentianinae was strongly supported (BS = 100%, PP = 1). Within Swertiinae, *Swertia* was polyphyletic in relation to the other genera (*Comastoma, Lomatogoniopsis, Lomatogonium, Halenia*, and *Veratrilla*) and distributed in four main clades. Besides, *Pterygocalyx volubilis* and two *Gentianopsis* consisted of a monophyletic basal clade, being sister to the four clades mentioned above. In Gentianinae lineage, three species of different genera formed a well-supported clade, being sister clade to Swertiinae (BS = 100%, PP = 1). Moreover, while most of these clades were distinct, however, *S. kouitchensis*, *S. punicea*, *S. franchetiana*, and *S. mussotii* clustered together in clade II with a near-zero branch length. These four species showed very low (0.0000683–0.0006831) pairwise distance values when compared to the overall mean pairwise distance (0.01) among all the 20 *Swertia* species (Additional file 8, Table S8). The same scenario was also found in clade IV, where *S. przewalskii*, *S. bifolia*, *S. souliei*, and *S. wolfangiana* clustered together with remarkably low pairwise distance values (0.0000929–0.0007623) (Additional file 8, Table S8). According to the pairwise distance calculation, the largest sequence divergence was found between *S. tetraptera* and *S. pubescens* (0.025819), and the lowest was between *S. franchetiana* and *S. mussotii* (0.0000683) (Additional file 8, Table S8).

### Selective pressure in plastid genes

The site-specific model showed none of the 50 CDSs of the 35 Gentianaceae species was under strong positive selection. Though, the *cemA* (ω = 0.59623) and *ycf1* (ω = 0.95807) were found under slightly positive selection. The ω ratios of the most genes were less than 0.2, indicating that they were under a strong purifying selection (Additional file 7, Table S7). The branch-specific model detected that the *cemA* was under positive selection in most *Swertia* clades along the phylogenetic tree (Fig. 8, Additional file 10, Fig. S2), including Clade I (ω = 290), Clade III (ω = 35) and Clade IV (ω = 999), but not for Clade II (ω < 1). For the *cemA*, the two-ratio model (m2) was significantly favoured over the one-ratio model (m0, 2ΔL = 54.96; *P* < 0.05). However, for the *ycf1*, both two-ratio model (m2) and free-ratio model (m1) were refused in the LRT test, indicating the same ω ratio of the *ycf1* for all branches in the phylogeny (Additional file 7, Table S7).

## Discussion

### Plastome structural evolution and likely adaptation of *Swertia*

The plastome sequences newly obtained from the 11 *Swertia* species, plus previously sequenced eight species (Addition file 1) were very similar in the overall structure, length, gene order, and contents (Table 1 and Fig. 1). However, the pseudogenes, especially ψ*rps16* and ψ*infA*, deserved closer scrutiny. The *rps16* was in a state flux with pseudogene in 16 species (*S. bifolia, S. cordata, S. diluta, S. erythrosticta, S. franchetiana, S. macrosperma, S. przewalskii, S punicea, S. wolfganiana, S. tetraptera, S. kouitchensis, S. leducii, S. multicaulis, S. mussotii, S. verticillifolia, S. souliei*) and entirely missing in the rest four species (*S. cincta, S. dichotoma, S. pubescens, S. hispidicalyx*). The pseudogene *rps16* was caused by loss of the second exon and part of the intron. Losses of plastid-encoded *rps16* have been reported across a wide range of seed plants [15]. Among Gentianaceae with published plastome sequences, the loss of *rps16* has been previously documented for members of variable genera [26–30]. We assumed that the loss of functionality of *rps16* might be a synapomorphy shared in the whole family of Gentianaceae, implying an ancient loss in the ancestor of Gentianaceae. The gene loss was likely resulted from the intracellular nuclear-coded *rps16* that transferred from mitochondria [31–33].

The *InfA* was identified to be pseudogene in 11 *Swertia* species. Among them, there was no start codon in 10 species (*S. cordata, S. bifolia, S. leducii, S. franchetiana, S. mussotii, S. hispidicalyx, S. verticillifolia, S. souliei, S. wolfganiana, S. erythrosticta*), but in *S. macrosperma*, a 59 bp-long-sequence has been deleted. The *infA* was also found to be pseudogene in seven Gentiana species [34], a sister clade of *Swertia* in Gentianaceae. In an intensive comparative research on the *infA*, the entirely missing and/or pseudogene have been reported occurred across 309 angiosperms, suggesting this gene has been transferred to the nucleus many times in different seed plants [35].

Mutation pressure on DNA sequences and natural selection are the two major factors that have been widely accepted to account for codon usage bias [36]. Our analysis of the 20 *Swertia* plastomes revealed a codon bias having A or U at the third position (Additional file 3, Fig. 3). In the absence of selection pressures, this A/U bias has increased the RSCU values for A/U ending codons. The fact that chloroplast genomes had similar biases of codon usage suggested that chloroplasts, in general, might have particular characteristics of codon usage [37], our finding was consistent with previous reports from many other chloroplast genomes [38–40]. The distribution plot of ENC vs. GC3 for the 20 *Swertia* plastomes showed all the plastomes lying very close in the Wright line [24] with an expected ENC value (49–52%), suggesting the codon usage of *Swertia* was mainly constrained by a GC mutation bias but not natural selection (Fig. 4).

Long repeat sequences of plastomes have been reported to play major roles in genomic rearrangements and sequence variations [41, 42]. However, our study showed no correlation between large repeats and rearrangement events. SSRs have been used widely in plant genomic and evolutionary studies because of their high amounts of variability within species (Table 6) [43, 44]. Our study showed the most abundant SSRs were A/T rich mononucleotide, which was consistent with previous reports that SSRs usually consisted of polyA or polyT repeats and rarely contain G or C repeats [45, 46].

Our selective pressure analyses of 50 common CDSs (> 300 bp) extracted from the 35 Gentianaceae plastomes only identified the *cemA* being positively selected. The *cemA* was found to encode an envelope membrane protein [47]. More recent researches revealed the disruption of this gene led to increased light sensitivity and impacted the CO_2_ transportation system in plants [48, 49]. Genes related to a specific environment are normally assumed to be under positive selection [48]. Previous studies suggested that the origin and speciation of Swertiinae occurred within 10 Ma (the Late Miocene), and *Swertia* within 4 Ma (the Pliocene to the Quaternary), possibly triggered by the uplift of the Tibetan Plateau and the climate change associated [6, 50, 51]. During Mid to Late Miocene, vast areas on earth had experienced a climate cooling transition [52, 53], its driven factor, as believed by many researchers, was the decline in atmospheric CO_2_ levels, which subsequently led to a transition of the modern ecosystem, e.g., the global radiation of C4 plants [54–58]. Therefore, the positive selection detected in *cemA* might indicate it occurred in the adaptation and speciation processes in *Swertia* species during the Late Miocene as a response to low level of atmospheric CO_2._ Interestingly, more positive selection was detected in species from higher-elevation regions in the Qing-Tibetan Plateau region of China (Clade IV) but no positive selection was found in species from lower-elevation regions (the eastern Qing-Tibetan Plateau and the south western China) (Clade II) (Additional file 10, Fig. S2), suggesting an adaptive response to the low atmospheric CO_2_ environment of the Qinghai-Tibetan Plateau region. However, more *Swertia* species are needed to be included to test this hypothesis whether positive selection detected on *cemA* as a beneficial result in the evolution of *Swertia* genus. In addition, further studies are needed to uncover whether changes in selection pressure are associated with specific biochemical pathways or functions of the *cemA* in the adaptation and divergence of *Swertia*.

### Candidate DNA barcodes

DNA barcodes are defined as short DNA sequences with adequate variations to identify species in a given taxonomic group [59]. Variable Loci (e.g., *matK*, *rbcL*, *trnH*-*psbA*) from plastomes have been widely used to determine the phylogeny of seed plants [59, 60]. Variable cpDNA loci have been employed to uncover phylogeny patterns of *Swertia*, including *trnL*-*UAA* intron, *trnL*-*UAA_trnF*-*GAA*, and *trnS*-*UGA_ycf9* [4], *matK* and *trnL*-*UAA* intron [5], *matK* [8], *matK* and *rbcL* [6]. However, these results were suffered from weak solutions. Furthermore, several clades were left unsolved *in* previously published phylogenetic trees. Therefore, additional loci are needed to be included to achieve higher phylogenetic reference of the genus *Swertia*. Moreover, loci used in these previous studies (e.g., *matK*, *trnL*-*UAA* intron, *rbcL*), are today considered intermediately variable regions [61]. Based on our nucleotide diversity analyses, only *trnL*-*UAA* intron had a high Pi value (ranked four), other loci had intermediate to low Pi values (e.g., *trnL*-*UAA_trnF*-*GAA* ranked 19, *matK* ranked 49, *rbcL* ranked 111) (Additional file 6, Table S6). Consequently, additional phylogenetically informative loci should be included to increase the phylogenetic resolutions of *Swertia*. Based on our study, we proposed a set of 10 most divergent loci *(trnH*-*GUG_psbA*, *ndhC_trnV*-*UAC*, *petB* intron, *trnL*-*UAA* intron, *rps16*, *rps16_trnK*-*UUU*, *trnS*-*UCC_trnG*-*UCC*, *trnS*-*GGA_rps4*, *psbM_trnD*-*GUC*, *ccsA_ndhD*) that be helpful for future phylogenetic and barcoding studies of *Swertia*.

### Phylogenetic analysis

Our plastome phylogenetic study did not produce any surprises topologically: the relationship inferred from the 74 common CDSs were consistent with clades resolved in previously published phylogenetic trees, *Swertia* was polyphyletic with its closely-related allies, namely V*eratrilla, Halenia*, *Comastoma, Lomatogonium*, and *Lomatogoniopsis* [4–6, 8, 9]. However, our study has greatly improved the phylogenetic resolution of the inferred clades in comparison with these earlier published trees, with most of the nodes having 100% support. The 20 *Swertia* species divided into four well-supported clades in the phylogenetic tree. Our molecular phylogeny study agreed with Ho TN taxonomic classification in sect*. Swertopsis* and sect*. Platynema* [1]*,* besides, a wide range of incongruences between morphological and molecular data was observed (Fig. 8).

Clade I comprised five species: two species belonging to subgen*. Poephila* (sect. Poephila and sect. Macranthos), paralleling three species of sect*. Platynema*, Subgen*. Ophilia*. Clade I was the sister clade to *Lomatogonipsis*-*Logmatogonium*-*Comastoma* lineage in a larger clade.

Clade II consisted of six species of sect*. Swertopsis*, subgen*. Ophilia*, forming a well-supported monophyletic clade. However, *S. leducii*, too, belonging to the sect*. Swertopsis*, was separated from Clade II and positioned in a larger group consisted of *Lomatogonipsis*-*Logmatogonium*-*Comastoma*, Clade I, and Clade II.

Clade III comprised two *Halenia* species, *S. dichotoma* of sect*. Japonicae*, subgen*. Ophelia*, and *S. tetraptera.* These four species clustered together with a notably higher solutions in internodes (BS 98–100%, 1.00 PP) than in previously published trees [6, 8], supported their close relationship. The taxonomic treatment of *S. tetraptera* has been in constant debates: Grisebach and Ma segregated it from *Swertia* as genus *Anagallidium* [62, 63]. Ho, based on morphology, pollination mode, and molecular evidence, segregated this species from *Swertia* and elevated it to a separate genus as *Sinoswertia,* but was not used in the recent account of the genus by Flora of China [2, 64]. Our phylogenetic study supported *S. tetraptera*’ close relationship with *S. dichotoma* (sect*. Japonicae*). Furthermore, these two *Swertia* species are more closely related to *Halenia* than with any other *Swertia* species. The genus *Halenia* is distributed in Asian mountains but with its diversification in south America [4, 5, 64], yet we only included two QTP-distributed *Halenia* species in this study, further studies are needed to discriminate the relationship between *Swertia* and *Halenia*.

Lastly, clade IV consisted of two paralleled groups: in one group, five species belonging to two different sections (sect*. Apterae* and sect*. Apterae*) of subgen*. Swertia* clustered together, being sister with *S. cordata,* a species from a different subgenus (sect*. Ophelia*, Subgen*. Ophilia*).

Our topology inferred from phylogenetic analyses included two clades that contained remarkably short terminal branches: *S. kouitchensis*-*S. punicea*-*S. fracnetiana*-*S. mussotii* in clade II and S. *przewalskii*-*S. bifolia*-*S. souliei*-*S. wolfganiana* in clade IV. This result indicated the emergence of these *Swertia* species might have occurred only recently, as confirmed by their extremely small genetic distance values, and also supported by our previous divergence time analysis based on *rbcL* and *matK* loci: most of the extant *Swertia* species were evolved during the Pliocene to the late Pleistocene period (4–0.1 Ma) [6]. Recent rapid radiations were detected in numerous studies, and can be explained by climate oscillations in the Quaternary [65–69]. Recently diverged species often have incomplete reproductive barriers and subsequently experience hybridization and/or introgression, especially where their habitats overlap [70]. On-going hybridization and introgression that accompanied recent rapid radiation have been detected in numerous studies, both plants and animals [71–75]. This could be the same scenario in *Swertia* and its allies in our study. Rapid radiations are among the most notorious phylogenetic problems, because retention and stochastic sorting of ancestral polymorphisms can yield incongruence between taxonomic circumscription and molecular data [76], as we observed in the case of *Swertia* and its closely related taxa, as well as in other rapidly diverged species [71–75]. To uncover the real evolutionary history of *Swertia*, we will use multi-locus molecular sequences that have evolved independently and population genomic approaches in our future study.

## Conclusion

This work was the first to report full sequence data and features of the whole plastomes of 11 *Swertia* species. The comparison of these plastomes revealed high similarities in terms of the overall structure, long repeat sequence, SSR, and codon usage. However, expansion and contraction in the IRb-LSC region were detected, due to the heterogeneous length of the ψ*ycf1*. Our phylogenetic analyses based on 74 CDSs supported the polyphyly of *Swertia*, was consistent with previous molecular studies, but with significant improved solutions. Furthermore, our phylogenetic study yielded extremely short branches, indicating a recent rapid radiation, hypothetically triggered by the Quaternary climate fluctuations. Our result inferred from the molecular data only partly supported the current taxonomic treatment. Furthermore, the *cemA* was identified under positive selection in most of the *Swertia* species, possibly reflecting an adaptive response to low CO_2_ atmospheric conditions during the Late Miocene. We proposed a set of 10 most variable loci *(trnH*-*GUG_psbA*, *ndhC_trnV*-*UAC*, *petB* intron, *trnL*-*UAA* intron, *rps16*, *rps16_trnK*-*UUU*, *trnS*-*UCC_trnG*-*UCC*, *trnS*-*GGA_rps4*, *psbM_trnD*-*GUC*, *ccsA_ndhD*) as markers for future phylogenetic and taxonomic studies of *Swertia*. Overall, our results demonstrated the power of plastome phylogenomic to improve phylogenetic resolution and contribute to a better understanding of plastid gene evolution in *Swertia*.

## Methods

### Sample collection, plastome sequencing, assembly, and annotation

We collected plant specimens in the Qing-Tibetan Plateau region (QTP) in China. Fresh leaves were collected in the field and dried in silica gel for the future DNA extraction. Total genomic DNA was extracted with the CTAB protocol [77]. Voucher specimens were deposited at the Herbarium of Northwest Institute of Plateau Biology, China Academy of Science (Xining, China) (Additional file 1, Table S1).

We sequenced the complete plastomes of the 11 *Swertia* species with an Illumina Hiseq 2500 sequencing system following instructions of DNA Nextera XT Sample Prep Kit (Illumina ™) at Novegene Co., Ltd. in Wuhan, China. Genomic DNA was fragmented randomly and then the required length of DNA fragments was obtained by electrophoresis. Adapters were ligated to DNA fragments followed by cluster preparation and sequencing. A paired-end library (150 × 2) was constructed with an insert size of 350 base pairs (bp), and then 150 bp paired reads were sequenced using the Illumina Hiseq 2500 sequencing system. The total number, length and quality of reads sequenced for each *Swertia* sample was analyzed with FastQC V. 0.11.9 [78]. Low-quality reads were filtered (> 50% of bases with a quality score ≤ 20 and ≥ 1% of missing bases) using SOAPnuke 2.1.0 [79]. High quality clean reads were assembled using NOVOPlasty v3.6 [80] with the default parameters. In order to validate the assembled plastome sequence error, the clean reads were mapped to the assembled plastomes using the Circlator V. 1.5.5 program [81]. The coverage depth of plastomes was calculated with the SAMtools V. 1.10 program [82]. Complete plastomes were annotated using the online program GeSeq [83] with two reference plastomes (*Swertia mussotii*, GenBank: KU641021 and *Gentianopsis grandis*, GenBank: NC_049879) using the default values to predict genes coding for proteins (CDS), transfer RNAs (tRNA), and ribosomal DNAs (rRNAs). The start and stop codon positions of the open reading frame for all the coding genes were manually checked and adjusted if necessary, with the Sequin program (NCBI). Organellar Genome DRAW [84] was employed to draw gene maps. The raw pair-end reads, the complete plastome sequence and gene annotation of the 11 newly assembled *Swertia* plastomes were submitted to the NCBI GenBank under BioProject No. PRJNA807092 (Additional file 1, Table S1).

### Plastome features

For each plastome, the length and gene were identified; LSC, SSC, IRa, and IRb region were plotted with junction positions being compared; the Guanine-cytosine (GC) content was calculated for each region with Geneious R7 [85].

### Codon usage

All CDSs for each *Swertia* plastome were extracted using Phylosuite v1.2.2 [86]. The amount of codon and relative synonymous codon usage (RSCU) ratio was calculated using Mega X [43]. The RSCU distribution was illustrated in the form of heatmaps using OriginPro2021 software (OriginLab Corporation, Northampton, MA, USA). The level of codon usage bias was determined by calculating ENC, GC1, GC2, GC3, and CAI with CodonW (http://codonw.sourceforge.net/). The ENC-GC3 (ENC vs. GC3) plot was carried out to examine whether the codon usage of a certain gene was affected by mutation or also by other factors such as natural selection. If the corresponding points were distributed around the expectation curve, it was possible to predict the mutation was the only factor affecting codon usage bias, as introduced by Wright [24] originally, and later improved by Liu [25]. The equation of the curve is as follows: pENC = 2.5—s + {29.5 / [s^2^ + (1 − s)^2^]}[24, 25]. The ENC vs. GC3 plot was generated by ggplot2 in R v.3.6.3 (https://www.r-project.org/).

### Characterization of repeat sequences and simple sequence repeats (SSR)

Forward, reverse, palindromic, and complementary repeats were identified by REPuter online program with the default settings, Maximum Computed Repeats 50 and Minimal Repeat Size 8 [87]. Simple sequence repeats (SSRs) were exploited using the MISA-web program [88] with the following parameters: ≥ 10 repeat units for mononucleotide SSRs, ≥ 6 for dinucleotide, and ≥ 3 for dinucleotide, tetranucleotide, pentanucleotide, and hexanucleotide SSRs. All overlapping repeats were removed from the final results.

### Hypervariable regions

For identifying hypervariable regions, the whole plastome alignment of the 20 *Swertia* plastomes was visualized using the mVISTA [89]. The CDSs, intergenic, and intron-containing regions of the 20 plastomes were extracted and aligned using Phylosuite v1.2.2 program [90]. Subsequently, the genetic divergence for all protein-coding and noncoding (intergenic spacers and introns) regions between the 20 *Swertia* plastomes was calculated based on nucleotide diversity value (Pi) using DnaSP v5.10.01 [91].

### Phylogenetic analysis

Phylogenetic analyses of *Swertia* were performed using both maximum likelihood (ML) and Bayesian inference (BI) methods based on the 74 CDSs shared between the 20 plastomes of *Swertia* (include eight published species) and altogether 11 species in subtribe Swertiinae and four species in Gentianaceae, with *Coffea arabica* (Rubiaceae) as the outgroup (Additional file 2, Table S2) [9, 92–99]. The nucleotide sequences of the common 74 CDSs were extracted from each plastome (with pseudogenes excluded), concatenated, and aligned with Phylosuite v1.2.2 [90], using default settings. The best‐fit models of nucleotide substitution, the GTR + I + G were selected by jModeltest v.2.1.7 [100] with the Akaike Information Criterion (AIC) [101]. The ML analyses were performed with RAXML v.8.2.12 [102]. Search for the best ML tree (the “‐f a” option) was using the GTRGAMMAI substitution model (GTR + I + G) that was selected by jModeltest, then drawing support values using the rapid bootstrap (1000 replicates). The BI analysis was performed for 20 million generations in MrBayes v.3.2.6 [103], with two runs of two independent Markov chain Monte Carlo (MCMC) chains. Each chain started with a random tree, default priors, and sampling trees every 100 generations, and the first 25% generation was discarded as burn‐in. Moreover, the evolution pairwise distance matrices were constructed with MEGA X based on the whole plastome sequences and 74 CDSs among the 20 *Swertia* species, using the Kimura 2-parameter model [43, 104].

### Selective pressure analysis

Selective pressures were analyzed for 50 common CDSs that are longer than 300 bp of 35 Gentianaceae species included in the phylogenetic analysis (see above). Firstly, the ratio (ω = dN/dS) of nonsynonymous (dN) to synonymous nucleotide (dS) substitution rates was calculated using the Codeml program in PAML4.9 with the site-specific model (seqtype = 1, model = 0, NSsites = 0, 1, 2, 3, 7, 8) [105, 106]. The codon frequencies were set by the F3 × 4 model. The likelihood ratio test (LRT) was used to identify positive selected sites in comparisons of M0 vs. M3, M1 vs. M2, M7 vs. M8. The Bayes Empirical Bayes (BEB) method was used to identify codons under positive selection. BEB values higher than 0.95 indicate sites that are potentially under positive selection. Furthermore, for CDSs that was detected under positive selection from the site-specific model, the branch model was used to detect signatures of positive selection along specific lineages by using the three models (one-ratio, free-ratio and two-ratio model). The one-ratio model (m0), assumes the same dN/dS ratio for all branches in the phylogeny. This model was compared to the free-ratio model (m1) that assumes an independent ω ratio for each branch. The one-ratio model (m0) was then compared to the two-ratio model (m2) that assumes the *Swertia* clades (set as the foreground branch) have ω ratios different from the other branches (set as the background branch). Similarly, the LRT was used to identify positive selected branches in comparisons of m0 vs. m1and m0 vs. m2 [105, 106].

## Supplementary Information

**Additional file 1:** **Table S1.** Basic information of 11 newly sequenced Swertia plastomes.

**Additional file 2:** **Table S2.** Information of species included in phylogenetic analyses.

**Additional file 3:** **Table S3.** RSCU values of protein-coding genes of the 20 Swertia plastomes.

**Additional file 4:** **Table S4.** Codon feature of the 20 Swertia plastomes.

**Additional file 5:** **Table S5.** The repeat sequence distribution in the 20 Swertia plastomes.

**Additional file 6:** **Table S6.** Information of the pairwise sequence divergence (Pi) of the 20 Swertia plastomes.

**Additional file 7:** **Table S7.** Results of selective pressure analysis.

**Additional file 8:** **Table S8.** Pairwise genetic distance of the 20 Swertia plastomes.

**Additional file 9:** **Figure S1.** Coverage depth of 11 newly sequenced Swertia plastomes.

**Additional file 10:** **Figure S2.** The ω ratio of cemA along the phylogenetic tree of Gentianaceae.

## Data Availability

The assembled sequences described in this study have been deposited in the National Center for Biotechnology and Information (NCBI) under the accessions as summarized in Additional File 1, Table S1.

## Abbreviations

BEB: Bayes empirical bayes
BI: Bayesian inference
bp: Base pair
BS: Branch support
CDS: Protein-coding sequence
CTAB: Cetyl trimethyl ammonium bromide
GC: Guanine-cytosine
IRs: Inverted repeats
ITS: Internal transcribed spacer
LRT: Likelihood ratio test
LSC: Large single copy
MCMC: Markov chain Monte Carlo
ML: Maximum likelihood
PP: Posterior probability
rRNA: Ribosomal RNA
RSCU: Relative synonymous codon usage
SSC: Small single copy
SSR: Simple sequence repeat
tRNA: Transfer RNA
