# Supplementary figures and images for: Plastome structure, phylogenomics and evolution of plastid genes in Swertia (Gentianaceae) in the Qing-Tibetan Plateau
Source: BMC Plant Biol. 2022 Apr 12;22:195. doi: 10.1186/s12870-022-03577-x (PMC9004202; doi:10.1186/s12870-022-03577-x)

Additional file 9:

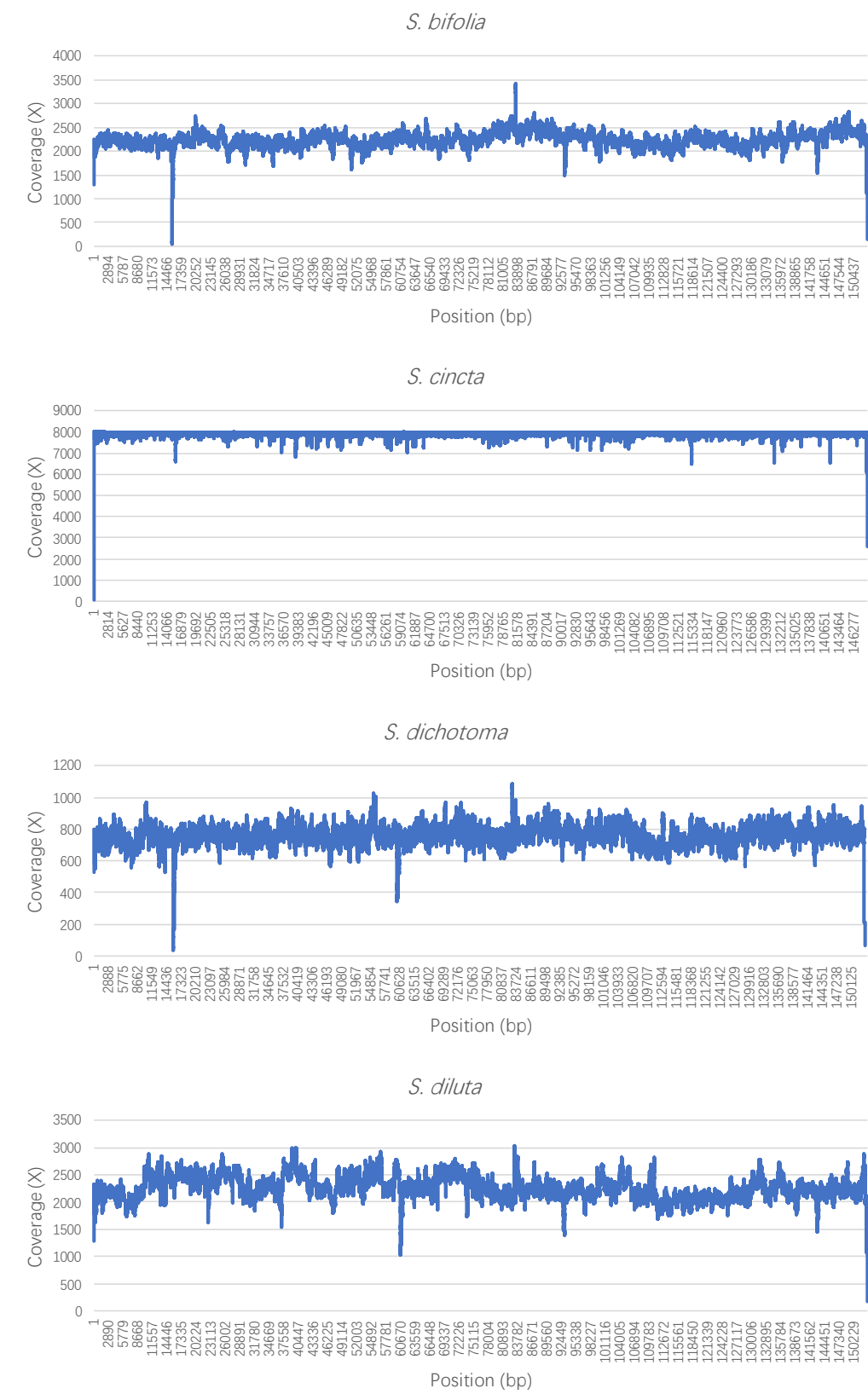

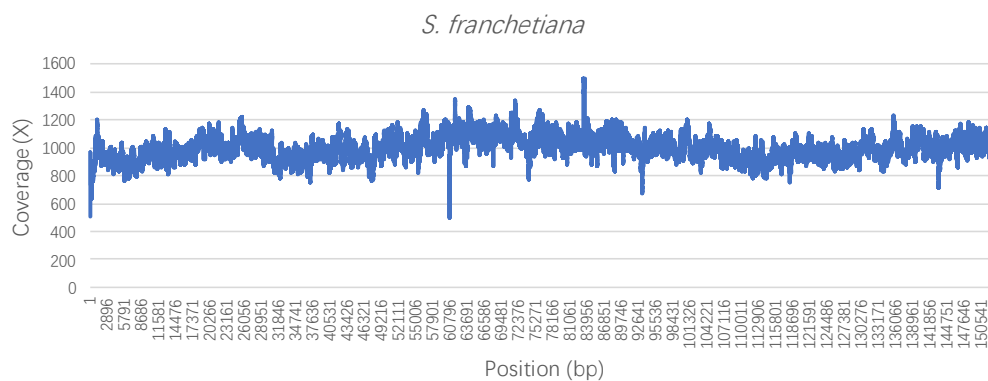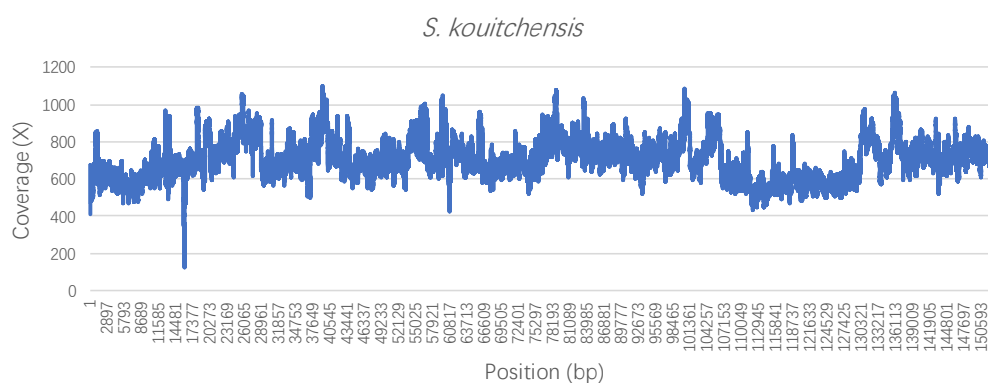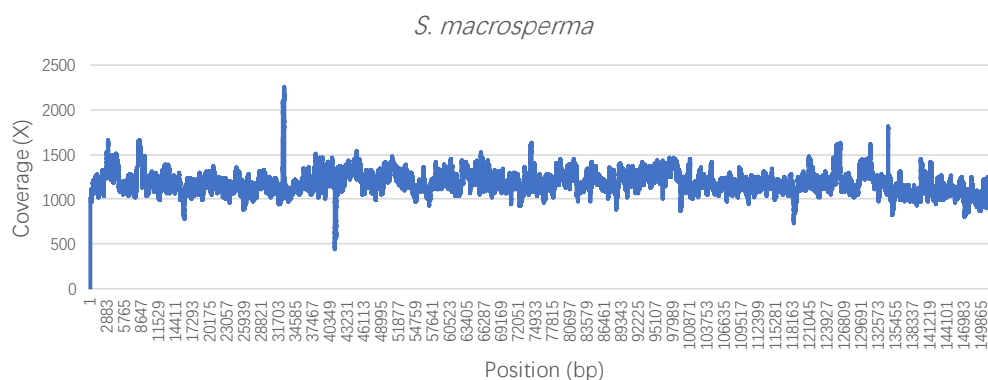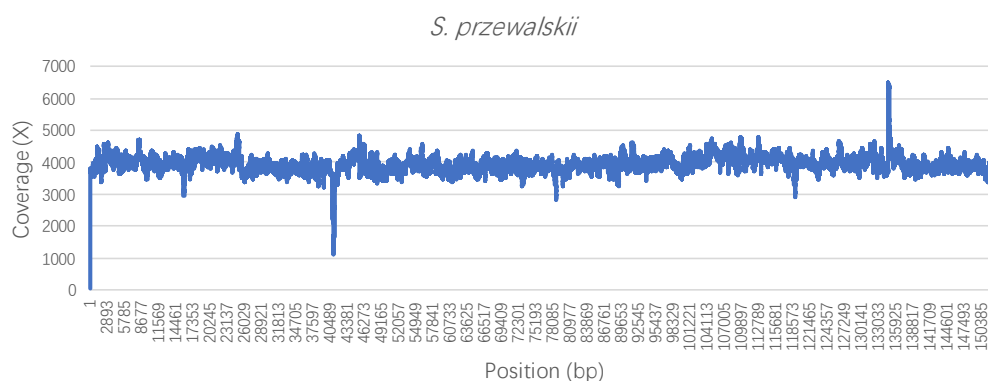

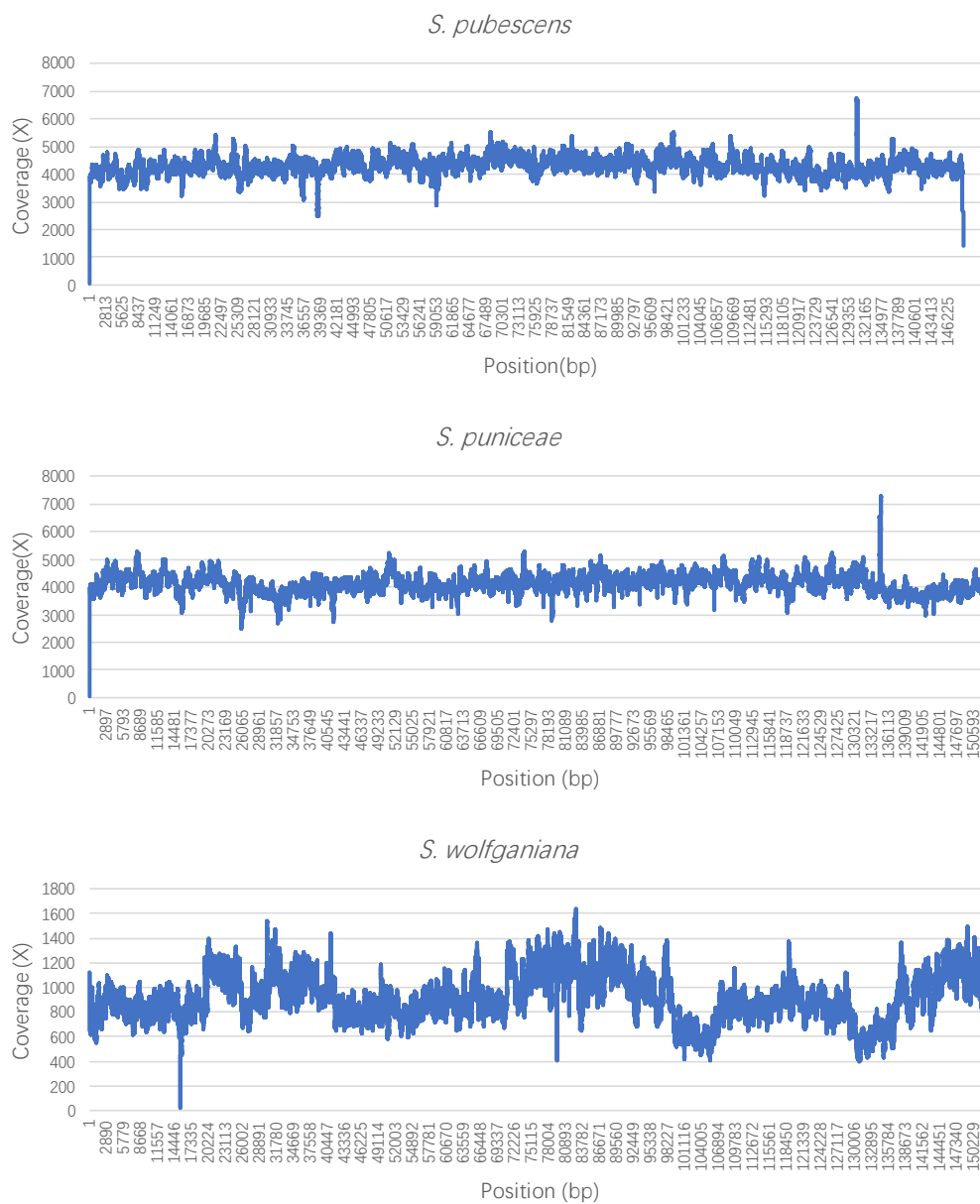

**Supplementary Figure S1.** Coverage depth of 11 newly sequenced *Swertia* plastomes.

Supplement: Supplementary file 9 — Additional file 9: Figure S1. Coverage depth of 11 newly sequenced Swertia plastomes. [file 12870_2022_3577_MOESM9_ESM.pdf]
