# Supplementary material for: Plastome structure, phylogenomics and evolution of plastid genes in Swertia (Gentianaceae) in the Qing-Tibetan Plateau
Source: BMC Plant Biol. 2022 Apr 12;22:195. doi: 10.1186/s12870-022-03577-x (PMC9004202; doi:10.1186/s12870-022-03577-x)
Supplement: Supplementary file 10 — Additional file 10: Figure S2. The ω ratio of cemA along the phylogenetic tree of Gentianaceae. [file 12870_2022_3577_MOESM10_ESM.pdf]

Additional file 10:

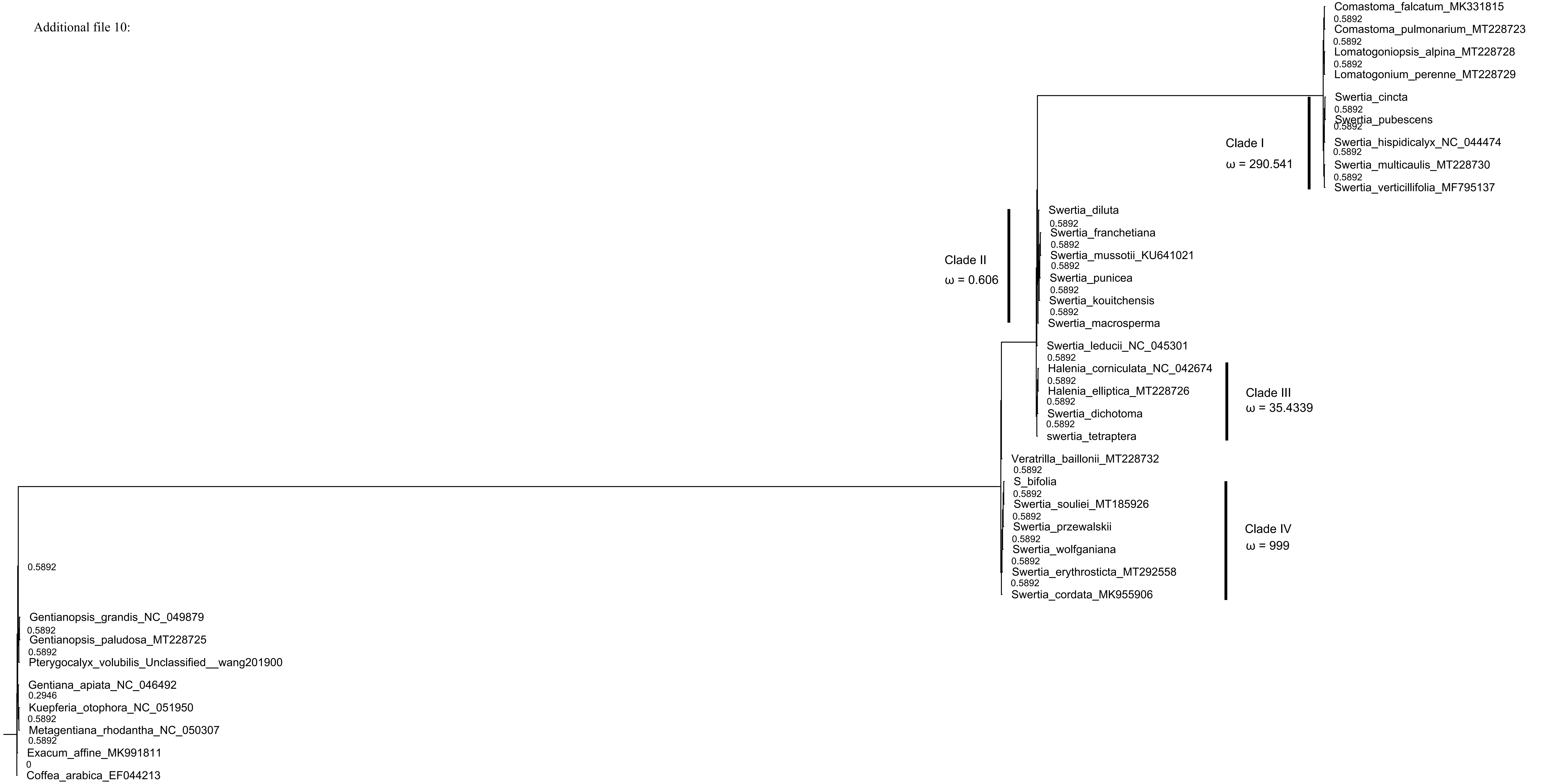

Supplementary Figure S2 The ω ratio of *cemA* along the phylogenetic tree of Gentianaceae
